# Supplementary material for: Web-browsing patterns reflect and shape mood and mental health
Source: Nat Hum Behav. 2024 Nov 21;9(1):133–46. doi: 10.1038/s41562-024-02065-6 (PMC11774758; doi:10.1038/s41562-024-02065-6)
Supplement: Supplementary file 1 — Supplementary Analyses 1–6, Tables 1–3, Fig. 1 and Materials. [file 41562_2024_2065_MOESM1_ESM.pdf]

nature human behaviour

Supplementary information https://doi.org/10.1038/s41562-024-02065-6

Web-browsing patterns reflect and shape mood and mental health

In the format provided by the authors and unedited

Supplementary

Supplementary Analysis 1

Fear sentiment of webpages browsed associated with mental health.

To test whether the specific emotions of webpages browsed were associated with mental health we quantified the percentage of *Anger, Fear, Anticipation, Trust, Surprise, Sadness, Joy* and *Disgust* associated words (as defined by the NRC Emotion Lexicon; Mohammad and Turney, 2013) out of all words on each webpage participants browsed. For each day separately, we then calculated the average emotion score of the webpages visited by each participant and then averaged these scores across the five days. We then input the eight Emotion scores, along with age and gender into a stepwise regression predicting the mean of the three psychopathology factors (i.e., '*Anxious-Depression*', '*Social-Withdrawal*' and '*Compulsive-Behaviour and Intrusive Thought*'). A stepwise regression was ideal in this case as the variance inflation factor (VIF) of some predictor variables was high (e.g., greater than 3). The winning model included the Fear score of webpages (**Study 1:** $\beta = 0.105 \pm 0.042$ (SE), $t(288) = 2.500$, $p = 0.013$, $r = 0.146$; **Study 2:** $\beta = 0.087 \pm 0.034$ (SE), $t(446) = 2.569$, $p = 0.011$, $r = 0.121$) as well as age and gender, suggesting that those with poorer mental health browse more fear related webpages. The Intraclass Correlation Coefficient (ICC) of the Fear scores across the 5 days revealed statistically significant moderate stability (ICC = 0.505, $p < 0.001$), indicating that the tendency to consume text high in fear words is relatively stable.

Supplementary Analysis 2

Average valence scores of webpages visited and affective ratings of webpages are not different when browsed on cell phones vs. laptops/desktops.

Twenty eight participants browse the internet for 15 minutes using a cell phone on one day and a desktop/laptop on the next. For each participant we then calculated the average valence score of the webpages browsed on each day. The valence scores were significantly correlated for information browsed on cell phones and lap/desktops (Negative: $r(26)= 0.641$, $p < 0.00$; Positive: $r(26)= 0.758$, $p < 0.001$) and the valence scores were not different across devices (frequentist paired-samples t-test results: for negative valence, Mean difference = -0.002, SD = 0.012, $t(27) = -0.672$, $p = 0.507$; for positive valence, Mean difference = 0.001, SD = 0.035, $t(27) = 0.158$, $p = 0.875$). A Bayesian paired-samples t-test supported these results, with Bayes factors of 0.24 for negative and 0.21 for positive valence scores, indicating moderate evidence for the stability of valence scores across device types. In other words, browsing on a phone is associated with browsing negative/positive content similarly to browsing on a laptop/desktop.

To explore whether participants evaluate the sentiment of webpages similarly across different devices (smartphones versus desktops/laptops), we conducted a study with a new group of 50 participants. Half of the participants were asked to rate the valence of the information on 24 webpages they visited on their smartphones using a 6-point Likert scale (ranging from 0, "not at all," to 6, "very much"). The questions focused on how positive or negative the information on the webpage was. The other half of the participants performed the same task, but on a desktop or laptop computer. We then examined the ratings of each of the 24 pages across the different devices. The results revealed a remarkably strong correlation in ratings regardless of whether the webpages were visited on a phone or desktop/laptop (positive scores: $r(23)= 0.980$, $p < 0.001$; negative scores: $r(23) = 0.995$, $p < 0.001$). This suggests that the emotional assessment of the webpages is similar, irrespective of the device used for browsing.

Together, these two sets of studies suggest that neither the valence of the webpages that are freely browsed, nor their emotional impact, alters across devices. Thus, while it is likely that people engage with their devices in different ways, the specific factors crucial for our analysis – the valence of the webpages that are freely browsed and their emotional impact – do not alter across devices

Supplementary Analysis 3

The valence scores of webpages show a strong positive correlation over time.

For our analysis text was extracted from webpages approximately 24-hours after participants visited them on average. As many webpages are non-static, we ran a validation study to check the stability of the valence scores of webpages. To that end, we assessed the valence of 1000 random webpages on day one and again the following day. Our findings revealed a significant positive correlation in both Negative scores ($r(998) = 0.912$, $p < 0.001$) and Positive scores ($r(998) = 0.902$, $p < 0.001$) across the two days. This suggests that the valence scores we

calculated based on text extracted on average 24-hours after participants visited the sites are very close to what we would have found if text was extracted immediately.

Supplementary Analysis 4

Effects of webpage valence on psychopathology and mood controlling for age, gender, income, ethnicity, education, language and excluding participants who visited password-protected social media webpages.

We examined the relationship between valence of webpages browsed and psychopathology, controlling for age, gender, income, ethnicity, education, language, and excluding participants who visited password-protected social media webpages. Negative Score of webpages browsed was associated with increased psychopathology symptoms [Study 1: $F(1,195) = 4.500$, $p = 0.035$, partial $\eta^2 = 0.023$, Study 2: $F(1,402) = 4.447$, $p = 0.036$, partial $\eta^2 = 0.011$] and with worse pre-browsing mood [Study 1, $t(77.82) = -2.361$, $p = 0.021$, $\beta = -0.118 \pm 0.050$ (SE); Study 2, $t(381) = -3.974$, $p = 0.047$, $\beta = -0.000 \pm 0.000$ (SE), partial $\eta^2 = 0.011$] and with worse post-browsing mood [Study 1, $t(38.09) = -2.604$, $p = 0.013$, $\beta = -0.055 \pm 0.021$ (SE); Study 2, $t(382) = -2.483$, $p = 0.013$, $\beta = -114.04 \pm 45.927$ (SE), partial $\eta^2 = 0.016$.] Positive Scores of webpages browsed were not associated with psychopathology symptoms in Study 1 [$F(1,195) = 0.006$, $p = 0.940$, partial $\eta^2 = 0.000$], but were in Study 2 [$F(1,402) = 6.154$, $p = 0.014$, partial $\eta^2 = 0.015$], neither were they associated with pre-browsing mood [Study 1, $t(75.82) = -0.459$, $p = 0.648$, $\beta = -0.024 \pm 0.052$ (SE); Study 2, $t(381) = 1.490$, $p = 0.137$, $\beta = -0.000 \pm 0.001$ (SE), partial $\eta^2 = 0.006$], nor post-browsing mood in Study 2 [$t(382) = 1.913$, $p = 0.057$, $\beta = 38.080 \pm 19.905$ (SE), partial $\eta^2 = 0.010$], but were associated with better post-browsing mood in Study 1 [$t(283.05) = 2.339$, $p = 0.020$, $\beta = 0.048 \pm 0.021$ (SE)].

Supplementary Table 1. *Demographics of Participants.*

Demographic	Study 1	Study 2	Study 3		Study 4a		Study 4b
			Negative Condition	Control Condition	Label Condition	Control Condition	
**Mean Age (SD)**	33.2 (11.7)	33.2 (12.0)	34.0 (9.7)	34.7 (12.1)	39.94 (13.68)	36.09 (9.97)	40.8 (12.9)
**Gender %**							
Male	48.1	41.8	49.1	51.1	33.3	47.3	40.5
Female	50.5	56.4	45.5	46.8	67.7	49.1	58.0
Other	1.4	1.8	5.5	2.1	0.0	3.6	1.5
**Ethnicity %**							
White	71.6	77.2	72.7	74.5	81.5	83.6	89.5
Black	5.2	4.9	9.1	10.6	3.7	7.3	3.0
Hispanic	1.4	2.5	9.1	0.0			0.5
Asian	18.3	12.5	3.6	8.5	9.3	7.3	4.5
Other	3.5	2.9	5.5	6.4	3.8	1.8	
**Income %**							
Under $5,000	23.3	14.3	12.7	19.1			8.5
$5,000 – $10,000	7.7	7.8	9.1	2.1			9.5
$10,001 – $15,000	12.2	11.7	9.1	4.3			7.5
$15,001 – $25,000	14.6	11.7	9.1	12.8			14.5
$25,001 – $35,000	16.0	11.7	7.3	12.8			17.0
$35,001 – $50,000	8.0	14.3	9.1	14.9			21.5
$50,001 – $65,000	10.5	7.8	12.7	10.6			10.0
$65,001 – $80,000	4.5	6.5	10.9	6.4			6.0
$80,001 – $100,000	1.7	6.5	10.9	6.4			2.5
Over $100,000	1.4	7.8	9.1	10.6			3.0
**Education %**							
High School Diploma	31.8	29.1	32.7	27.7			30.5

2- Year Degree	8.7	11.0	16.4	12.8			11.5
4-Year Degree	28.7	27.5	34.5	38.3			33.5
Postgraduate/Professional Degree	23.2	26.0	16.4	21.3			20.0
Other	7.6	6.5	0.0	0.0			4.5
**First Language %**							
English	84.0	88.3	100.0	100.0	92.6	89.1	94.5
Other	16.0	11.7	0.0	0.0	7.4	10.9	5.5

Supplementary Table 2. ***Studies 3 and 4a. Comparing the demographics across experimental groups.*** *(Groups were not different on any demographic variables in either study 3 or 4).*

Variable	Study 3	Study 4a
**Age**	t(100) = 0.351, p = 0.726	t(94.90) = -1.668, p = 0.099
**Gender**	$\chi^2(2, 102) = 0.745$, p = 0.689	$\chi^2(2, 109) = 4.731$, p = 0.094
**Ethnicity**	$\chi^2(4, 102) = 5.406$, p = 0.248	$\chi^2(6, 109) = 4.813$, p = 0.568
**Education**	t(100) = 0.842, p = 0.402	NA
**Income**	t(100) = -0.361, p = 0.719	NA
**Language**	NA (all participants first language is English)	$\chi^2(2, 109) = 2.001$, p = 0.368
		(All tests are two-sided)

Supplementary Analysis 5

Study 3, 4 a&b: controlling for demographics:

Controlling for the demographics reported in Table 2 and for pre-browsing mood: post browsing mood (β = -13.105 ± 2.694 (SE), t(101) = -4.865, p = 0.001, partial eta^2 = 0.208) was worse in the negative valence condition than the control condition, in Study 3. Controlling for the demographics reported in Table 3, participants in the label condition browsed more positive labels (β = 0.364 ± 0.182 (SE), t(102) = 3.179, p = 0.048, partial eta^2 = 0.040) and less negative labels (t(102) = -2.451, trend p = 0.064, partial eta^2 = 0.035) than the no-label condition, with no differences in browsing neutral webpages (β = -0.044 ± 0.178 (SE, t(102) = -0.249, p = 0.804, partial eta^2 = 0.000) in Study 4a. Controlling for the demographics reported in Table 3, valence of label (-1 = Negative, 0 = Neutral, 1 = Positive) was positively associated with post browsing mood (β = 0.092 ± 0.022 (SE), t(160.74) = 3.947, p = 0.001) controlling for pre-browsing mood in study 4b.

Supplementary Table 3. *The impact of Age and Gender on psychopathology scores and browsing patters (Study 1 and 2).*

Full Model	Age ß	Gender ß
**Study 1: (Mean Psychopathology ~ Negative score + Age + Gender)**	-0.179*** (i.e., younger participants report more psychopathology symptoms).	0.132** (i.e., females report more psychopathology symptoms).
**Study 2: (Mean Psychopathology ~ Negative score + Age + Gender)**	-0.135*** (i.e., younger participants report more psychopathology symptoms).	0.122*** (i.e., females report more psychopathology symptoms).
**Study 1: (Negative score ~ Pre-Mood + Age + Gender)**	0.008* (i.e., older participants browse more negative webpages).	-0.141(not significant).

**Study 2: (Negative score ~ Pre-Mood + Age + Gender)**	-0.071 (not significant).	-0.177*** (i.e., males browse more negative webpages).
**Study 1: (Post Mood ~ Negative score + Pre-Mood + Age + Gender)**	0.000 (not significant).	-0.080** (i.e., males report better mood after browsing the web).
**Study 2: (Post Mood ~ Negative score + Pre-Mood + Age + Gender)**	-0.029 (not significant).	-0.070* (i.e., males report better mood after browsing the web).
***** = P <0.001, ** = P <0.01 * = P <0.05. (two-sided), not corrected.**		

<u>Supplementary Analysis 6</u>

Results from Studies 1 & 2 using the Hu & Liu Valence lexicon (2004).

We implemented the exact same approach described within but this time with a different valence lexicon (Hu & Liu, 2004), which categorizes 2006 words as positive and 4783 as negative. Other than that, the score for positive words and negative words were calculated exactly as described in the main text. Note, the Hu and Liu lexicon does not categorize specific emotions, so analysis was only conducted using its Negative and Positive scores.

We examined the relationship between valence of webpages browsed and psychopathology controlling for age and gender. Negative Score of webpages browsed was associated with increased psychopathology symptoms [Study 1: $F(1,284) = 4.083$, $p = 0.044$, partial $\eta^2 = 0.014$, Study 2: $F(1,442) = 6.462$, $p = 0.011$, partial $\eta^2 = 0.014$] and with worse pre-browsing mood in Study 2 [$F(1,442) = 4.168$, $p = 0.042$, partial $\eta^2 = 0.009$] and a trend in Study 1 [$t(364.49) = -1.720$, $p = 0.086$, $\beta = -0.071 \pm 0.041$]. and with worse post-browsing mood [Study 1:$t(715.58) = -1.977$, $p = 0.048$, $\beta = -0.035 \pm 0.018$ (SE); Study 2, $t(399) = -2.244$, $p = 0.025$, $\beta = -1.413 \pm 0.630$ (SE)]. Positive Scores of webpages browsed were not associated with psychopathology symptoms in Study 1 [$F(1,284) = 0.155$, $p = 0.695$, partial $\eta^2 = 0.001$], but were in Study 2 [$t(399) = -2.293$, $p = 0.022$, $\beta = -0.001 \pm 0.000$ (SE)], neither were they associated with pre-browsing mood [Study 1, $t(75.82) = -0.459$, $p = 0.648$, $\beta = -0.024 \pm 0.052$ (SE); Study 2, $t(381) = 1.490$, $p = 0.137$, $\beta = -0.000 \pm 0.001$ (SE), partial $\eta^2 = 0.006$], nor post-browsing mood [Study 1, $t(94.11) = -0.723$, $p = 0.471$, $\beta = -0.029 \pm 0.041$ (SE); Study 2, $t(399) = 0.861$, $p = 0.390$, $\beta = -0.000 \pm 0.001$ (SE)].

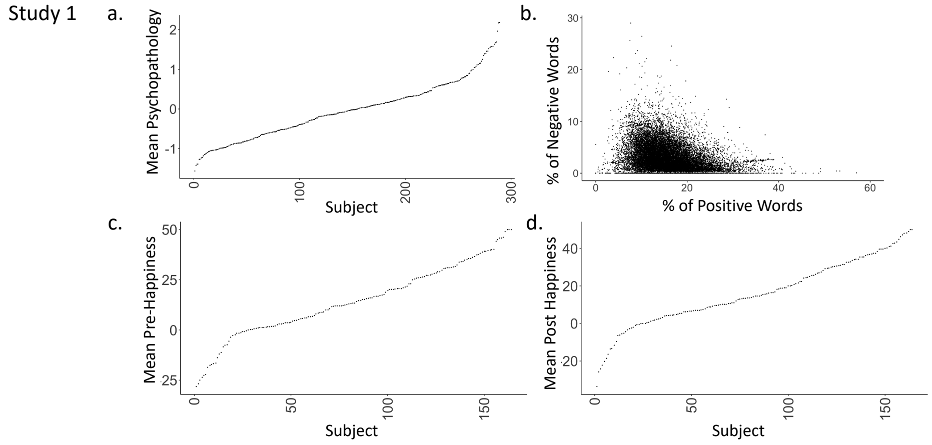

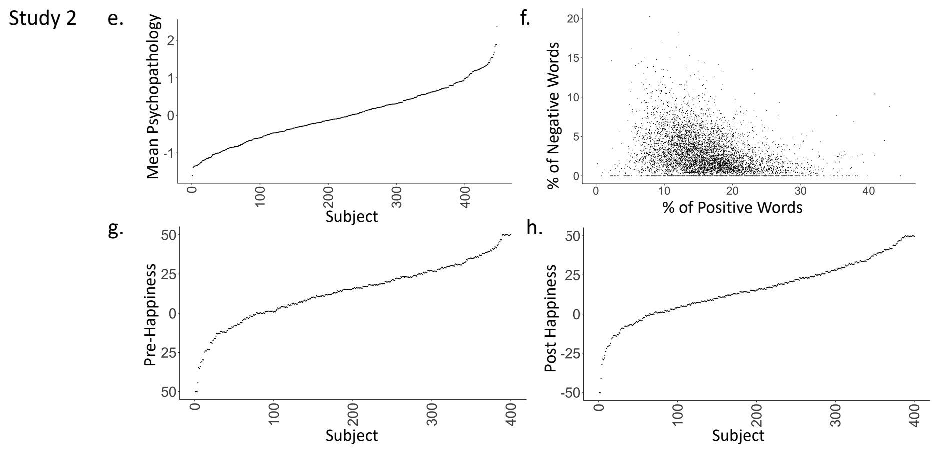

Supplementary Figure. (a,e) Plotted are the mean psychopathology scores on the y-axis for participants on the x-axis in **(a)** Study 1 and **(e)** Study 2. Each dot represents an individual's score, sequenced from the smallest (indicative of lower psychopathology symptoms) to the largest (indicative of higher psychopathology symptoms). **(b,f)** Plotted are the Positive (on the x-axis) and Negative (on the y-axis) scores for the webpages viewed by participants in **(b)** Study 1 and **(f)** Study 2, calculated using the NRC method. Each dot represents a webpage. **(c,d,g,h)** Plotted are the mean **(c)** pre-happiness and **(d)** post-happiness scores (y-axis) for participants (x-axis) in Study 1, as well as **(g)** pre-happiness and **(h)** post-happiness scores (y-axis) for participants (x-axis) in Study 2. Each dot represents an individual's score, sequenced from the smallest (less happy) to the largest (more happy).

Supplementary Materials

Study Instructions (Studies 1-4)

In Study 1, participants were introduced to the study with the following statement: *"In this study, we will ask you to search the internet for 20 minutes a day for 5 days. Once you complete the first session, you will then be invited each day for the following 4 days to complete another session. Only on the first day will we ask you to complete questionnaires about your demographics and wellbeing."*

In Study 2 participants were introduced to the study with the following statement: *"In this study, we will ask you to search the internet for 30 minutes and to complete questionnaires about your demographics and wellbeing."*

In Study 3 participants were introduced to the study with the following statement: *"Now, you will be asked to read the information on two webpages. Afterwards, you will be asked questions about each webpages you read."*

Participants were also instructed the following in Studies 1, 2 and 3:
"1 Please browse the internet during your free time (i.e. not during work hours).
2. Please do not visit any sites which require a password, e.g. social media, Netflix, Prolific, Amazon MTurk or bank websites etc. Also we require that you do not watch videos during this time, as we cannot analyse this data."

In Study 4, participants were introduced to the study with the following statement: *"In this study, you will be shown a list of webpages that come up when you search for something on Google. Each webpage will have a score next to it that shows how the information on that webpage will likely make you feel (for example, it might make you feel worse, neutral, or better). You will be asked to choose one of the webpages to visit and read all the information on it. To do this, you will need to copy and paste the link for the webpage into a new tab on your internet browser. Next, we will ask you a question about the information you read. You will do this for 6 times."*
