# Supplementary material for: Web-browsing patterns reflect and shape mood and mental health
Source: Nat Hum Behav. 2024 Nov 21;9(1):133–46. doi: 10.1038/s41562-024-02065-6 (PMC11774758; doi:10.1038/s41562-024-02065-6)
Supplement: Supplementary file 2 — Reporting Summary [file 41562_2024_2065_MOESM2_ESM.pdf]

nature portfolio

Corresponding author(s): Christopher Kelly & Tali Sharot

Last updated by author(s): Oct 10, 2024

# Reporting Summary

Nature Portfolio wishes to improve the reproducibility of the work that we publish. This form provides structure for consistency and transparency in reporting. For further information on Nature Portfolio policies, see our Editorial Policies and the Editorial Policy Checklist.

## Statistics

For all statistical analyses, confirm that the following items are present in the figure legend, table legend, main text, or Methods section.

| n/a | Confirmed | |
|---|---|---|
| ☐ | ☒ | The exact sample size (*n*) for each experimental group/condition, given as a discrete number and unit of measurement |
| ☐ | ☒ | A statement on whether measurements were taken from distinct samples or whether the same sample was measured repeatedly |
| ☐ | ☒ | The statistical test(s) used AND whether they are one- or two-sided<br>*Only common tests should be described solely by name; describe more complex techniques in the Methods section.* |
| ☐ | ☒ | A description of all covariates tested |
| ☐ | ☒ | A description of any assumptions or corrections, such as tests of normality and adjustment for multiple comparisons |
| ☐ | ☒ | A full description of the statistical parameters including central tendency (e.g. means) or other basic estimates (e.g. regression coefficient) AND variation (e.g. standard deviation) or associated estimates of uncertainty (e.g. confidence intervals) |
| ☐ | ☒ | For null hypothesis testing, the test statistic (e.g. $F$, $t$, $r$) with confidence intervals, effect sizes, degrees of freedom and $P$ value noted<br>*Give P values as exact values whenever suitable.* |
| ☒ | ☐ | For Bayesian analysis, information on the choice of priors and Markov chain Monte Carlo settings |
| ☒ | ☐ | For hierarchical and complex designs, identification of the appropriate level for tests and full reporting of outcomes |
| ☐ | ☒ | Estimates of effect sizes (e.g. Cohen's *d*, Pearson's *r*), indicating how they were calculated |

*Our web collection on statistics for biologists contains articles on many of the points above.*

## Software and code

Policy information about availability of computer code

| | |
|---|---|
| Data collection | The experiments were designed using Qualtrics' online survey platform. Participants were recruited using Prolific. Participants were asked to browse the web using Firefox. |
| Data analysis | IBM SPSS 27, R studio Version 2022.12.0+353, Google Colab (i.e., Python). |

For manuscripts utilizing custom algorithms or software that are central to the research but not yet described in published literature, software must be made available to editors and reviewers. We strongly encourage code deposition in a community repository (e.g. GitHub). See the Nature Portfolio guidelines for submitting code & software for further information.

## Data

Policy information about availability of data

All manuscripts must include a data availability statement. This statement should provide the following information, where applicable:

- Accession codes, unique identifiers, or web links for publicly available datasets
- A description of any restrictions on data availability
- For clinical datasets or third party data, please ensure that the statement adheres to our policy

Data and Code availability: Anonymized data and code are available at a dedicated repository [https://github.com/affective-brain-lab/WebbrowsingNHB].

## Research involving human participants, their data, or biological material

Policy information about studies with human participants or human data. See also policy information about sex, gender (identity/presentation), and sexual orientation and race, ethnicity and racism.

| | |
|---|---|
| Reporting on sex and gender | We only inquired about Gender. Necessary information has been reported in the manuscript and below. |
| Reporting on race, ethnicity, or other socially relevant groupings | In addition to gender we report on age, ethnicity, income, education and language. |
| Population characteristics | See below. |
| Recruitment | Participants were recruited using Prolific's online recruitment platforms. The purpose of this study was not mentioned in the advertisement of the study. |
| Ethics oversight | Studies were approved by the ethics committee at UCL and all subjects gave informed consent. |

Note that full information on the approval of the study protocol must also be provided in the manuscript.

# Field-specific reporting

Please select the one below that is the best fit for your research. If you are not sure, read the appropriate sections before making your selection.

☐ Life sciences ☒ Behavioural & social sciences ☐ Ecological, evolutionary & environmental sciences

For a reference copy of the document with all sections, see nature.com/documents/nr-reporting-summary-flat.pdf

# Behavioural & social sciences study design

All studies must disclose on these points even when the disclosure is negative.

| | |
|---|---|
| Study description | This studies investigated the relationship between web-browsing behavior and well-being. Study 1 was a longitudinal study, while Study 2 was a cross-sectional study. Studies 3-4 were between-groups studies The data is analysed using quantitative methods. |
| Research sample | Our research sample included participants recruited from Prolific: Study 1 (N = 289, age = 33.17, SD =11.71; females = 50.5%, males = 48.1%, other = 1.4%), Study 2 (N = 447, age = 33.85, SD =12.58; females = 56.4%, males = 41.8%, other = 1.8% ), Study 3 (negative valence condition: N = 55, age=33.96, SD=9.68; females=45.5%, males = 49.1%, other = 5.5%; control condition:N = 47, age=34.72, SD=12.14; females=46.8%, males = 51.1%, other = 2.1%), Study 4a (label condition: N = 55; no label condition: N = 54, Study 4b (IN = 200, age = 40.8, SD = 12.9; females = 58.0%, males = 50.5%, other, 1.5%). This allowed us to recruit a large random sample of participants. Our sample is not necessarily representative of the general population. |
| Sampling strategy | For studies 1 and 2, sample size was calculated based on a pilot study.<br>For studies 3 and 4a&b, sample size was checked post-hoc for power.<br>Power analysis for studies 1-4 were conducted using G*power (http://www.psychologie.hhu.de/arbeitsgruppen/allgemeinepsychologie-und-arbeitspsychologie/gpower.html), with 1-beta = .80 and alpha = 0.05.<br>All studies implemented a convenient sampling strategy (online participants). |
| Data collection | All data was collected online using Qualtrics' survey platform by Christopher Kelly. All data was anonymised. |
| Timing | Study 1: 28/11/2020 - 26/02/2021<br>Study 2: 22/03/2021 - 11/3/2022<br>Study 3: 15/08/2022<br>Study 4a: 22/11/2022<br>Study 4b: 14/11/2023 |
| Data exclusions | Study 1: 23 participants from whom we could not obtain at least 1KB of text from a minimum of 3 webpages a day were not analyzed.<br>Study 2: 53 participants from whom we could not obtain at least 1KB of text from a minimum of 3 webpages a day were not analyzed.<br>Study 3: 37 participants from whom we could not obtain at least 1KB of text from a minimum of 3 webpages a day were not analyzed.<br>Study 4a: No participants were excluded.<br>Study 4b: No participants were excluded. |
| Non-participation | No participants dropped out/declined participation. |
| Randomization | There are no groups/conditions to be randomized in Studies 1,2,4b. In Studies 3 and 4a, participants were not randomly assigned to different conditions; instead, they unknowingly self-assigned, with conditions conducted within 35-minutes (Study 1) and 120-minutes (Study 2) of each other. The study instructions were identical across the respective study conditions, ensuring participant were not aware to which conditions they were signing up to, nor that there was conditions. |

# Reporting for specific materials, systems and methods

We require information from authors about some types of materials, experimental systems and methods used in many studies. Here, indicate whether each material, system or method listed is relevant to your study. If you are not sure if a list item applies to your research, read the appropriate section before selecting a response.

## Materials & experimental systems

| n/a | Involved in the study |
| --- | --- |
| ☒ | ☐ Antibodies |
| ☒ | ☐ Eukaryotic cell lines |
| ☒ | ☐ Palaeontology and archaeology |
| ☒ | ☐ Animals and other organisms |
| ☒ | ☐ Clinical data |
| ☒ | ☐ Dual use research of concern |
| ☒ | ☐ Plants |

## Methods

| n/a | Involved in the study |
| --- | --- |
| ☒ | ☐ ChIP-seq |
| ☒ | ☐ Flow cytometry |
| ☒ | ☐ MRI-based neuroimaging |
